# Podocalyxin in the onset of nephropathy among Indian type 2 diabetes mellitus patients

**DOI:** 10.6026/973206300191124

**Published:** 2023-12-31

**Authors:** Balaji Viswanatha Setty, Raghav Gutlur Nagarajaiah Setty, Hareesh Rangaswamaiah, Ganesh Veluri

**Affiliations:** 1 Department of General Medicine, Rajarajeshwari Medical College and Hospital, Bangalore, Karnataka, India; 2 Department of General Medicine, Rajarajeshwari Medical College and Hospital, Bangalore, Karnataka , India; 3 Department of General Medicine, Akash Institute of Medical Sciences and Research Centre, Bangalore, Karnataka, India; 4 Department of Biochemistry, ESIC Medical College and Hospital, Kalaburagi, Karnataka, India

**Keywords:** Type 2 diabetes mellitus, nephropathy, microalbumin, podocalyxin

## Abstract

The diabetic nephropathy is one of the most prevalent microvascular complications with type 2 diabetes mellitus. The most accurate and widely used marker for diabetic nephropathy is microalbuminuria and it is also regarded as conventional method. However, it is not a sensitive or specific nephropathy biomarker. Therefore, it is of interest to evaluate the role of podocalyxin to predict early onset of nephropathy in patients with type 2 diabetes mellitus. This cross - sectional study is conducted on 150 subjects. Among these 150 T2DM patients (Group 2: T2DM with normoalbuminuria and Group 3: T2DM with microalbuminuria) and 50 were age, gender and BMI matched healthy controls (Group 1). The biochemical and experimental parameters was analyzed. T2DM patients have higher levels of urine podocalyxin. This level was significantly elevated in patients with T2DM with microalbuminuria than normoalbuminuria. Urinary podocalyxin levels and HbA1c were found to be positively correlated. Thus, urinary podocalyxin is useful as early predictable marker for nephropathy in patients with type 2 diabetes mellitus.

## Background:

The complex condition known as type 2 diabetes mellitus (T2DM) is defined by hyperglycemia brought on by decreased insulin secretion by pancreatic beta cells as well as insulin resistance in the body's target tissues, including the liver and skeletal muscle [1]. The metabolic derangements in T2DM lead to secondary complications that affect multiple organ systems, including a greater predisposition to cardiac disease. DM is the third leading cause of death (after heart disease and cancer) in many developed countries [2-3]. The complications of diabetes affect mainly the eye, kidney and nervous system which lead to blindness, renal failure, amputation, heart attacks and stroke [4]. Hyperglycemia is the major cause for these complications in T2DM. The international diabetic federation stated 425 million people between the ages of 20 and 79 were afflicted by 2017, that figure is predicted to rise to 629 million by 2045 [5]. However, in Indian scenario, in 2017, 77 million people are living with T2DM and this number expecting to reach 139 million by 2045 [6]. Glomerular hyperfiltration, thickening of the glomerular basement membrane, and an expansion of extracellular matrix in mesangial zones are only a few examples of the structural and functional alterations in the glomerulus that lead to diabetic nephropathy (DN) [7]. Recent research has shown that proximal tubular cell atrophy and tubulo-interstitial fibrosis is just as critical as glomerulosclerosis in terms of the prognosis of the kidneys. Currently, micro albuminuria is regarded as the gold standard and the earliest clinically available sign for nephropathy identification. However, the main drawbacks of micro albuminuria were low sensitivity and greater unpredictability. Even a significant fraction of renal impairment starts out as normo-albuminuria or occurs before micro albuminuria develops [8]. A few studies have also shown that diabetic people can still acquire DN even in the absence of protein excretion, and that some patients with severe DN may relapse to normo-albuminuria. Exercise, food, smoking, obesity, illness, and inflammation are additional circumstances that cause protein to be lost through urine [9].

Podocalyxin (PCX) is a transmembrane O-glycosylated and sialylated protein that is mostly found in podocytes and is also expressed in neurons, vascular endothelium, and hematopoietic progenitor cells [10]. This may be seen on the podocytes' apical surface and forms a meshwork that supports the glomerulus' capillaries. It can also be seen expressing laterally between cells and in the intercellular spaces between the podocytes, and the inter digitating foot process creates a slit diaphragm [11]. Damage to podocytes can result from a number of clinical diseases, and as a result, podocalyxin is excreted in urine. The results of this urinary PCX test reveal the severity of the damage to the glomerular epithelial cells [12]. Urinary podocalyxin (Urinary PCX), according to recent studies, was employed as a measure for the degree of active glomerular damage and a predictor of disease progression. Therefore, it is of interest to evaluate the role of podocalyxin to predict early onset of nephropathy in patients with type 2 diabetes mellitus.

## Materials and Methods:

A cross-sectional study was carried out in the department of medicine, Rajarajeshwari Medical College and Hospital, Bangalore, Karnataka from 2021 and 2023. The American Diabetic Association (ADA) [13] Criteria were used to diagnose 150 individuals with T2DM who were treated at the General Medicine OPD. According to kidney disease improve global outcomes (KDIGO) criteria [14], The degree of albuminuria was used to divide the diabetic individuals into two groups. The microalbumin is less than 30 mg/g creatinine is considered normoalbuminuria; between 30 and 300 mg/g creatinine is considered microalbuminuria. As controls, 50 volunteers were chosen whose age, gender, and Body Mass Index (BMI) matched those of healthy people. Institutional Ethics Committee (IEC) at Rajarajeshwari Medical College and Hospital authorized the study. After receiving informed consent, all of the study's participants were enrolled. All participants in the trial had to be between the ages of 30 and 70, and T2DM patients with various stages of nephropathy identified using ADA and Kidney Disease Improving Global Outcomes (KDIGO) criteria were included. Patients with macrovascular complications like cardiovascular, cerebrovascular, and peripheral vascular diseases, active inflammatory disease, urinary tract infections, people taking thiazolidinediones, anti-inflammatory were excluded from the study. After 10-12 hours of overnight fasting, 6 mL of venous blood samples were taken from each participant in the study. Six (6) mL of blood were divided into three tubes: 2 ml transferred to tube containing sodium fluoride, another 2ml transferred to containing ethylene diamine tetra-acetic acid (EDTA), and the last tube containing the remaining 2 mL was plain. After two hours following breakfast, 2 mL of venous blood was once more drawn from the same subjects. Plasma and serum were separated from the blood samples by centrifugation at 3000 rpm for 10 minutes and stored until biochemical analysis was done. Along with the blood sample, a spot urine sample was also collected. Urinary albumin and creatinine were immediately analyzed after the urine sample was centrifuged at 3000 rpm for 10 minutes. Later, 1 mL of urine transferred appropriate label aliquots. The fasting blood sugar (FBS), post prandial blood sugar (PPBS), serum urea, serum creatinine, and microalbumin was measured by using laboratory standard methods. The eGFR was calculated by modified diet in renal diseases (MDRD) formula. The urinary podocalyxin was measured by using enzyme linked immunosorbent assay.

## Statistical analysis:

Kolmogorov-Smirnov test was used to assess the distribution of the data and data was expressed mean and standard deviation (SD). ANOVA was used to assess differences between the three groups under investigation, and it was followed by post hoc multiple testing using Tamhane's or Bonferroni's tests, if necessary. The correlations between the markers were examined using Pearson's correlation analysis. Microsoft Excel and SPSS were used for the statistical analysis and P value is <0.05 was considered statistically significant.

## Results:

The mean and standard deviation of FBS, PPBS in the controls, T2DM patients with normo and microalbuminuria was found to be elevated, the P value is <0.05. The urea and creatinine concentrations were significantly elevated T2DM patients with microalbuminuria when compared to T2DM patients with normoalbuminuria and controls, P value is 0.05. The glycated hemoglobin was significantly elevated in both the groups of T2DM patients when compared to healthy controls (P<0.05). Urinary podocalyxin levels are significantly higher in T2DM patients with normo and microalbuminuria when compared to healthy controls, (P<0.05). Table 1

Comparison of biochemical parameters and urinary markers between the groups was studied using Bonferroni's or Tamhane's multiple comparison tests and tabulated in Table 2. All the parameters were showed in between the groups (P<0.05) except creatinine does not shown significance between group 1 and group 2, respectively P value is 0.790.

Pearson's correlation analysis of the study parameters showed in Table 3. All the parameters was significantly positive correlation with urinary podocalyxin, the P value is less than 0.05. The urinary podocalyxin was showed a direct association with nephropathy in patients with T2DM.

**Figure 1:**
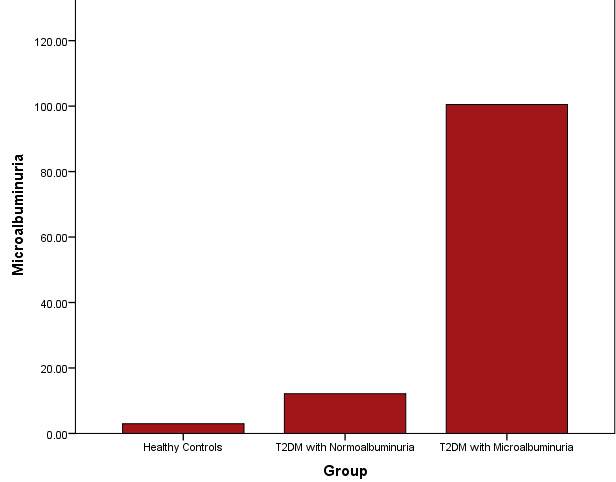
Distribution of microalbumin concentrations among study subjects

Figure 1 showed the distribution of microalbumin concentrations between the study subjects. There was a significantly elevated levels of microalbumin levels in T2DM patients with microalbuminuria when T2DM patients with normoalbuminuria, and healthy controls.

**Figure 2:**
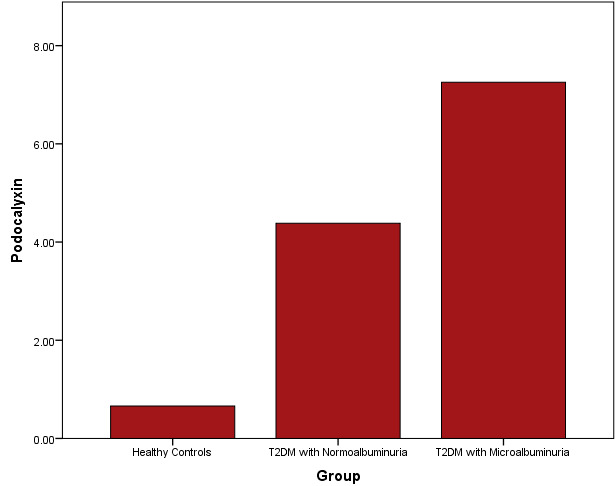
Distribution of podocalyxin concentrations among study subjects

Figure 2 showed the distribution of podocalyxin concentrations between the study subjects. There was a significantly elevated levels of podocalyxin levels in T2DM patients with microalbuminuria when T2DM patients with normoalbuminuria, and healthy controls.

**Figure 3:**
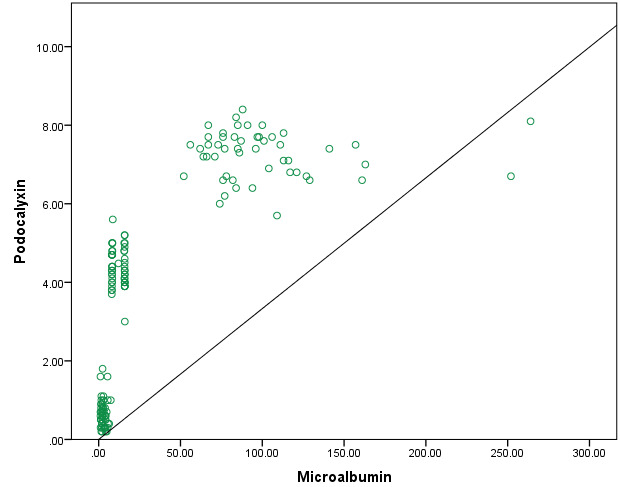
Scatter plots between podocalyxin and microalbumin among study subjects

Figure 3 showed the scatter plots between podocalyxin and microalbumin concentrations. There was a significantly direct association between podocalyxin and microalbumin levels, respectively P value is less than 0.05.

## Discussion:

A couple of the main causes of ESRD and one of the most common conditions worldwide is diabetic nephropathy. Persistent microalbuminuria (30-300 mg/day) proven on at least 2 occasions at intervals of 3-6 months is currently the gold standard test for the early diagnosis of diabetic nephropathy. But it has drawbacks, including a limited sensitivity, a plasma concentration that is influenced by a number of variables, and a large inter-individual variance of 47% [15- 16]. To lessen the burden of chronic renal disease in T2DM, innovative biomarkers for the early detection of DN and progression to ESRD must be discovered. Hence, the present study aimed to evaluate the role of podocalyxin to predict early onset of nephropathy in patients with type 2 diabetes mellitus. In the present study, the mean ± SD of FBS in the controls, T2DM patients with normoalbuminuria and T2DM patients with microalbuminuria was found to be increasing respectively; p<0.001). This is an expected finding considering the inclusion criteria of the subjects into these groups. The mean ± SD of serum urea levels and serum creatinine showed an increasing trend among the groups studied (p<0.001), although they were within the reference range in all the groups. In the present study, Urinary albumin creatinine ratio (UACR) in the controls, T2DM patients with normoalbuminuria and T2DM patients with microalbuminuria, respectively showing a statistically significant increase across the groups (p<0.001).

The first DN sign now used in clinical practice is microalbuminuria. However, a significant amount of renal damage happens before microalbuminuria develops or in a non-albuminuric condition. Hence there is a need for identification of novel biomarker for early diagnosis of DN. Accordingly the present study estimated urinary podocalyxin in urine of healthy controls as well as T2DM patients with normoalbuminuria and microalbuminuria. The glomerular filtration barrier is normally made up of the podocyte and foot process, glomerular basement membrane, and capillary endothelial cells. The glomerular filtration system's performance may be impacted by disruption to this filtration barrier. It was discovered that urine indicators such Podocalyxin may be used to diagnose kidney impairment in DN patients [17]. Monitoring the amount of podocyte cells in the urine or estimating podocyte urinary biomarkers can both be used to assess podocyte damage. In the present study also urinary podocalyxin levels were found be increased in patients with T2DM with normoalbuminuria (p=0.001**) as well as microalbuminuria (p <0.001**) when compared to controls. Also, the increase in microalbuminuria diabetic patients was more when compared to the normoalbuminuric counterparts (p=0.017).

Urinary podocalyxin is a vesicle-like substance that develops on the apical surface of podocytes. Patients with microalbuminuria in DM patients had greater levels of podocalyxin than those with normoalbuminuria [18]. Podocalyxin levels in the urine were greater in 53.8% of patients with normoalbuminuria, 64.7% of patients with microalbuminuria, and 66.7% of patients with macroalbuminuria, suggesting that urinary Podocalyxin may be a helpful biomarker for spotting early podocyte impairment in diabetic patients [19]. Unexpectedly, higher urinary podocalyxin levels were seen in 53.8% of normoalbuminuric patients, suggesting that urinary podocalyxin may serve as a valuable biomarker for spotting early podocyte damage in diabetes patients.

In a similar manner, another study that included 142 patients with glomerular disorders, 71 people with T2DM, and 69 healthy volunteers found that PCX detection in urine indicates damage to the apical region of podocytes and can be utilized as a marker for the early stages of nephropathy in people with T2DM. In addition, 45 healthy controls were included in a cross-sectional study that comprised 116 T2DM patients who were divided into normo-albuminuria, micro-albuminuria, and macro-albuminuria [20]. According to the study, all T2DM patient categories had significantly higher urine PCX levels, which were favorably connected with albuminuria. The consequently, urinary PCX might be thought of as one of the early, reliable indicators of DN.

## Conclusion:

Data shows that urinary podocalyxin was significantly increased in all the subgroups of T2DM patients. Thus, the urinary podocalyxin is useful as early predictable marker for nephropathy in patients with type 2 diabetes mellitus.

## Figures and Tables

**Table 1:** Comparison of biochemical and clinical characteristics in between the groups

**Parameters**		**Mean**	**Std. deviation**	**P-Value**
Age	Healthy Controls	45.8	7.12	0.001**
	T2DM with Normoalbuminuria	51.64	5.96	
	T2DM with Microalbuminuria	56.18	7.47	
FBS	Healthy Controls	97.62	8.3	0.001**
	T2DM with Normoalbuminuria	159.48	13.1	
	T2DM with Microalbuminuria	186.76	57.33	
PPBS	Healthy Controls	118.06	15.72	0.001**
	T2DM with Normoalbuminuria	196.26	11.47	
	T2DM with Microalbuminuria	279.22	63.65	
Urea	Healthy Controls	19.16	4.81	0.001**
	T2DM with Normoalbuminuria	29.8	3.07	
	T2DM with Microalbuminuria	84.64	7.66	
Creatinine	Healthy Controls	0.74	0.15	0.001**
	T2DM with Normoalbuminuria	0.71	0.2	
	T2DM with Microalbuminuria	4.43	0.49	
HbA1c	Healthy Controls	4.05	0.58	0.001**
	T2DM with Normoalbuminuria	7.55	0.74	
	T2DM with Microalbuminuria	8.66	0.39	
Microalbumin	Healthy Controls	2.97	1.57	0.001**
	T2DM with Normoalbuminuria	12.11	3.83	
	T2DM with Microalbuminuria	100.48	41.62	
Podocalyxin	Healthy Controls	0.66	0.36	0.001**
	T2DM with Normoalbuminuria	4.38	0.48	

**Table 2:** Comparison of variables in between the study subjects

**Parameter**	**Group 1 vs Group 2**	**Group 1 vs Group 3**	**Group 2 vs Group 3**
FBS	0.001**	0.001**	0.005*
PPBS	0.001**	0.001**	0.001**
Urea	0.001**	0.001**	0.001**
Creatinine	0.79	0.001**	0.001**
HbA1c	0.001**	0.001**	0.001**
Microalbumin	0.001**	0.001**	0.001**
Podocalyxin	0.001**	0.001**	0.001**

**Table 3:** Correlation of variables between the study subjects

Parameter		FBS	PPBS	**Urea**	**Creatinine**	**HbA1C**	**Microalbumin**	**Podocalyxin**
FBS	Pearson Correlation	1	.658**	.592**	.537**	.703**	.545**	.701**
	Sig. (2-tailed)		0	0	0	0	0	0
PPBS	Pearson Correlation	.658**	1	.783**	.759**	.791**	.706**	.848**
	Sig. (2-tailed)	0		0	0	0	0	0
Urea	Pearson Correlation	.592**	.783**	1	.955**	.750**	.852**	.870**
	Sig. (2-tailed)	0	0		0	0	0	0
Creatinine	Pearson Correlation	.537**	.759**	.955**	1	.639**	.853**	.800**
	Sig. (2-tailed)	0	0	0		0	0	0
HbA1c	Pearson Correlation	.703**	.791**	.750**	.639**	1	.635**	.920**
	Sig. (2-tailed)	0	0	0	0		0	0
Microalbumin	Pearson Correlation	.545**	.706**	.852**	.853**	.635**	1	.750**
	Sig. (2-tailed)	0	0	0	0	0		0
Podocalyxin	Pearson Correlation	.701**	.848**	.870**	.800**	.920**	.750**	1
	Sig. (2-tailed)	0	0	0	0	0	0	

## References

[1] Wang R (2020). Biomed Res Int.

[2] Akankwasa G (2018). Biomark Med.

[3] National Kidney Foundation. (2012). Am J Kidney Dis.

[4] Tung CW (2018). Nephrology.

[5] Petrica L (2017). J Diabetes Complications.

[6] Ozdemir AM (2005). Ann N Y Acad Sci.

[7] Petrica L (2023). Int J Mol Sci.

[8] Cellesi F (2015). Curr Opin Nephrol Hypertens.

[9] Ye H (2014). J Diabetes Complications.

[10] Shoji M (2016). Biomarkers.

[11] Ikuma D (2018). Lupus.

[12] Asao R (2012). Clin J Am Soc Nephrol.

[13] Huang D (2012). Ban.

[14] Hara M (2012). Diabetologia.

[15] Asao R (2012). Clin J Am Soc Nephrol.

[16] Kostovska I (2020). Rom J Intern Med.

[17] Shoji M (2016). Biomarkers.

[18] Suwanpen C (2016). J Nephrol.

[19] El-Ashmawy HM (2019). J Diabetes Complications.

[20] Wang R (2020). Biomed Res Int.

